# Synthesis, crystal structure, photoluminescence and catalytic properties of a novel cuprous complex with 2,3-pyrazinedicarboxylic acid ligands

**DOI:** 10.1038/s41598-020-63277-0

**Published:** 2020-04-14

**Authors:** Xingxing Zheng, Yanmei Chen, Jingwen Ran, Li Li

**Affiliations:** Hubei Key Laboratory for Processing and Application of Catalytic Materials, College of Chemistry and Chemical Engineering, Huanggang Normal University, Huanggang, 438000 China

**Keywords:** Environmental sciences, Pollution remediation, Chemistry

## Abstract

A copper (I) polymer, [Cu_2_Mg(pzdc)_2_(H_2_O)_5_•2H_2_O]_n_ (pzdc = 2,3-Pyrazinedicarboxylic acid), was synthesized by solvothermal method. The complex was characterized using X-ray diffraction (XRD), fourier transform infrared spectroscopy (FT-IR), ultraviolet-visible spectrophotometry (UV-Vis), and element analysis. X-ray crystal structure analysis reveals that the complex is a two-dimensional coordination polymer. The photophysical and luminescent properties of the complex were investigated. At room temperature, the complex exhibits reversible double strands cyclic voltammogram and displays efficient blue emission with peak maxima at 468 nm. Catalytic liquid phase oxidation of dyes and glyphosate was carried out using the copper (I) polymer as catalyst and H_2_O_2_ as oxidant. Degradation efficiencies were evaluated by varying the reaction conditions (i.e. illumination and oxidant). In the degradation reactions, the polymer exhibits high degradation efficiency within a short reaction time under the optimum reaction conditions. Furthermore, the reusability of the catalyst is excellent, showing no activity loss in four repeated cycles. The possible reaction mechanism catalyzed by the polymer was inferred on the basis of the results of electron spin resonance (ESR), electrochemical and ion chromatography analyses (IC).

## Introduction

Coordination polymers have received considerable attention because of their unique characteristics in terms of magnetic behaviors, optical activities as well as catalytic and luminescent properties. Polycarboxylate ligands were widely studied owing to their rich coordination modes and desired topologies^1–3^. Complexes with phthalic acid, pyromellitic acid^4–8^ and heterocyclic polycarboxylic acid^9–13^ ligands were characterized and investigated. For example, 2,3-pyrazinedicarboxylic acid ligand was involved in the design and synthesis of a large number of Cu and Cu-Ln complexes with novel structures and properties^14–19^. Acting as polyfunctional ligand in metal complexes, pyrazine-2,3-dicarboxylate ligand coordinated with transition metal ions using its four carboxylate oxygen atoms and two nitrogen atoms. However, from the structures of the metal complexes, the N and O atoms are not fully utilized in coordination, employing only half or even less for the formation of the complexes. Nonetheless, with the change of reaction condition this traditional ligand could form new types of polymers. Herein, we report the synthesis of a novel Cu-based polymer using simple 2,3-pyrazinedicarboxylic acid as ligand. Based on the polymer structure, it is deduced that five out of the six carboxylate atoms participate in the coordination.

Cu-based complexes have attracted much attention because of their luminescent and emission properties, and are widely used in various fields. It is known that their properties are governed by not only the ligand structures but also the steric effect imposed by the ligands^20–23^. Generally, Cu (I) complexes with luminescent property can be divided into acetylide clusters, cuprous halide clusters^24^, trinuclear pyrazolate complexes^25,26^, and mononuclear Cu (I) complexes. For these complexes, a diimine and a diphosphane ligand or two diimine ligands are commonly involved in the coordination. It should be emphasized that the copper (I) complex of the present study is a two-dimensional (2D) polymer with luminescent behavior. In addition, copper-based complexes have been used in a wide range of catalytic reactions^27–29^. With the facile change of Cu oxidation state, the Cu-based complexes can directly drive or catalyze many redox reactions. Also, because the complexes are insoluble, they can be easily separated and reused. The oxidation processes are eco-friendly because H_2_O_2_ is commonly used as oxidant, with water being the only by-product. In this article, we report the outstanding photocatalytic performance of the copper (I) polymer in the degradation of dyes and glyphosate, and conduct discussion on the related mechanism.

## Experimental

### Materials and methods

Reagent grade 2,3-pyrazinedicarboxylic acid, oxalyl dihydrazide, copper nitrate trihydrate, Mg(OH)_2_, hydrogen peroxide (30% solution), glyphosate, rhodamine B (RhB), methyl orange (MO) were all purchased from Shanghai sinopharm chemical reagent Co. Ltd. They were used without any further purification. In the studies, purified water was used as solvent.

The elemental analysis of C, H and N was performed on a model 2400 Perkin-Elmer analyzer. The IR spectra were recorded (KBr pellets) on a Nicolet 170SX spectrophotometer in the 4000–400 cm^−1^ region. The UV-Vis absorption spectra were recorded (BaSO_4_ pellets) on a SHIMADZU UV-2600 UV-Vis spectrophotometer in the range of 200–700 nm. Powder X-ray diffraction (PXRD) measurements were performed at room temperature on a Philips X’pert MPD Pro X-ray diffractometer using Cu Kα radiation (λ = 0.154 18 nm), with the X-ray tube operated at 40 kV and 40 mA. The single crystal X-ray diffraction (SXRD) data were performed in Jilin University. The IC analyses were recorded on an 883 Basic IC plus-Metrohm, and the emission spectra on a SHIMADZU RF-5301pc fluorescence spectrophotometer. The ESR results were obtained in the Institute of Chemistry, Chinese Academic of Sciences. Electrochemical measurement was performed on a CHI-660 instrument. Total organic carbon (TOC) measurement was performed on a TOC-L CPN instrument(SHIMADZU).

For the photocatalytic experiments, a Xe lamp (300 W) (from Jingyuan Co. Ltd., Beijing) was used to provide the visible light. To eliminate ultraviolet (UV) light and ensure visible light (λ > 420 nm) irradiation, a cutoff filter (3 cm in diameter) was placed in front of the lamp to completely remove the light of wavelengths less than 420 nm^30^. The light intensity at the sample surface was 38 mW/cm^2^. RhB, MO and glyphosate were selected as pollutants for degradation at room temperature. Prior to illumination, the suspension containing 80 mg of catalyst and 100 mL of dye or glyphosate solution (20 mg L^−1^) was stirred for 30 min in the dark for adsorption–desorption equilibrium. Then 1 mL of H_2_O_2_ was added to the suspension and the Xe lamp was turned on for the start of degradation. At given time intervals, 2 mL of suspension was collected with a syringe and treated by needle tube filtration (0.45 μm, PVDF membrane) and analyzed. For dye degradation, the analysis was by means of UV/Vis spectrophotometry whereas for glyphosate degradation, it was by TOC and IC methods.

### Synthesis of copper (I) complex

The reaction was carried out in a 10 mL high pressure resistant glass bottle under autogenous pressure. The reactor with pyrazine-2, 3-dicarboxylic acid (1 mmol, 168 mg), Mg(OH)_2_ (1 mmol, 58 mg), copper nitrate trihydrate (0.5 mmol, 121 mg), oxalyl dihydrazide (1 mmol, 118 mg) and H_2_O (5 mL) was sealed and heated at 120 °C for 24 h before cooling to room temperature. The brown cubic crystals were collected by filtration, washed with purified water and ethanol and dried in air at room temperature. The product yield was 67% based on Cu(II): Anal. Calcd C_12_H_18_Cu_2_MgN_4_O_15_: C 23.64, H 2.98, N 9.19. Found: C 23.56, H 3.03, and N 9.25. IR (cm^−1^): 3601(vs), 2923(w), 1597(m), 1542(s), 1315(w), 1267(m), 954(m), 853(s), 525(m), 471(vs). In the FT-IR spectra of the complex, a high intensity broad band observed in the range of 3700–3500 cm^−1^ is attributed to OH stretching of the H_2_O. A medium intensity band in the range of 1625–1600 cm^−1^ is attributed to CN stretching which shifts lower to 1597–1542 cm^−1^ after coordination. This is further supported by the appearance of a medium intensity band in the range of 525–520 cm^−1^ which is assigned to Cu-N stretching^31^. The absence of strong characteristic peaks around 1720 cm^−1^ indicates that all carboxylate groups are completely deprotonated^32^.

### X-ray crystallography

The data were collected with Mo Kα radiation (λ= 0.71073 Å) on a Siemens SMART CCD diffractometer for the copper (I) complex. The structure was solved using direct methods with the SHELXTL program and refined using SHELXL-2016 by full-matrix least-squares techniques, the non-hydrogen atoms were assigned with aniso-tropic displacement parameters in the refinement, while the hydrogen atoms were treated using a riding model. The water H atoms were placed in calculated positions with O–H equal to 0.82 Å, and with torsion angles refined, Uiso (H) = 1.5Ueq(C, O). Aromatic H atoms were placed in calculated positions with C–H equal to 0.93(Å) and refined with Uiso (H) = 1.2Ueq(C). The copper (I) complex has been assigned the deposition number CCDC No. 1949198 and the data can be obtained free of charge from the Cambridge Crystallographic Data Centre via http://www.ccdc.cam.ac.uk/data request/cif. The crystal data, data collection and structure refinement details are summarized in Table 1.

**Table 1:** Summary of crystal data, data collection and structure refinement for the complex.

Empirical formula	[C_12_H_18_Cu_2_MgN_4_O_15_]_n_
Formula weight	609.69n
Temperature/K	148(2)
Crystal system	monoclinic
Space group	P2_1_/c
a/Å	12.7868(5)
b/Å	8.4766(4)
c/Å	19.6854(9)
α/°	90
β/°	104.542(2)
γ/°	90
Volume/Å^3^	2065.32(16)
Z	4
ρ_calc_ g/cm^3^	1.961
μ/mm^−1^	2.177
F(000)	1232.0
Crystal size/mm^3^	0.28 × 0.25 × 0.19
Radiation	MoKα (λ = 0.71073)
2θ range for data collection/°	5.826 to 54.97
Index ranges	−16 ≤ h ≤ 15, −10 ≤ k ≤ 10, −25 ≤ l ≤ 25
Reflections collected	26397
Independent reflections	4596 [R_int_ = 0.0396, R_sigma_ = 0.0282]
Data/restraints/parameters	4596/21/349
Goodness-of-fit on F^2^	1.049
Final R indexes [I > = 2σ (I)]	R_1_ = 0.0374, wR_2_ = 0.0976
Final R indexes [all data]	R_1_ = 0.0587, wR_2_ = 0.1143
Largest diff. peak/hole / e Å^−3^	1.90/−0.54

## Results and Discussion

### Synthesis and structure

The copper (I) complex was *in situ* synthesized by hydrothermal method. During the reaction, oxalyl dihydrazide decomposed into hydrazine and oxalate upon heating. Then hydrazine reduced copper (II) to cuprous ions while oxalate reacted with Mg^2+^ to form colorless magnesium oxalate dehydrate. The cuprous ions coordinated with pyrazine 2,3-dicarboxylate while Mg^2+^ participated in coordination as counterbalance ions to form the copper (I) complex. We attempted to obtain the target product by replacing oxalyl dihydrazide with hydrazine hydrate or ascorbic acid, and Mg(OH)_2_ with MgCl_2_ but failed. It is referred that both oxalyl dihydrazide and Mg(OH)_2_ play an important role in the self-assembly process. Furthermore, it was observed that the redox reaction is a slow process and cuprous ion in diluted concentration is beneficial to the growth of single crystals.

**Figure 1:**
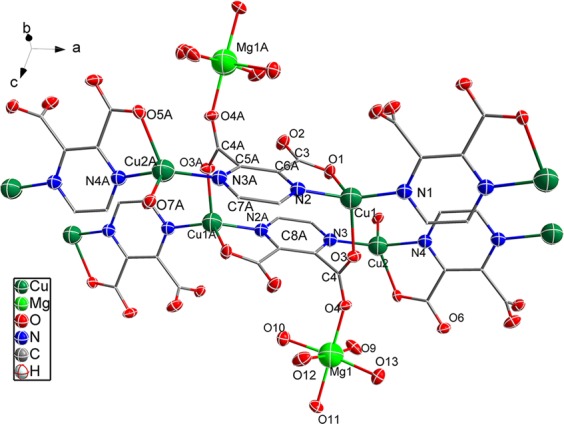
Crystal structure of copper (I) complex (**A**: 1-X, 1-Y, -Z). Hydrogen atoms and solvent molecules are omitted for clarity.

The crystal structure of the copper (I) complex is shown in Fig. 1. The complex crystallizes into a monoclinic system with P21/c space group. The central Cu1 ion coordinates with two carboxyl oxygen atoms (O1 and O3) and two nitrogen atoms (N1 and N2) originating from three pzdc^2−^ ligands, forming a distorted tetrahedron. The two N donors from the same pzdc^2−^ coordinate with two different Cu^+^ ions from the sides of pyrazine ring, forming a chain that extends into 1D space. The carboxyl group C3O1O2 (O1) coordinates to the Cu1 ion in a monodentate mode. The other carboxyl group C4AO3AO4A coordinates to Cu1A and Mg1A simultaneously (where symmetry code A is 1-X, 1-Y, -Z). The Mg^2+^ ion coordinates with the oxygen atoms of five water molecules as well as a carbonyl group oxygen atom from pzdc^2−^, forming a distorted octahedral geometry. Also, two Cu^+^ ions and two pzdc^2−^ form a [Cu_2_(pzdc)_2_] basic unit with distorted chair ring structure, and such a unit extends into 1D space with the pyrazine rings in parallel arrangement. Each Mg^2+^ shares a carbonyl oxygen atom from pzdc^2−^ and decorated the diagonal angle of the unit like an antennae. Each pzdc^2−^ provided five coordination atoms (N2, N3, O1, O3 and O4).

Selected bond lengths, angles and hydrogen bonds are given in Table 2. The Cu–O and Cu–N bond distance ranges from 2.156(2) Å to 2.176(2) Å and from 1.942(2) to 1.978(2), respectively. The data are different from those of reported Cu–O(pzdc^2−^) (1.942 Å, 1.980 Å) and Cu–N(pzdc^2−^) (2.023 Å) distances, with the former obviously longer and the latter a little shorter. As for the N − Cu–O bond angles, they are in the scope of 79.83(9)° to 120.81(9)°. These phenomena can be attributed to the different sizes of the central Cu ions (Cu^2+^(d^9^) and Cu^+^(d^10^)) as well as to Jahn-Teller effect.

**Table 2:** Selected bond lengths, angles and hydrogen bonds of copper (I) complex.

Atom–Atom	Length/Å	D H A	d(D-A)/Å
Cu1 N1	1.942(2)	O14 H14A O3	2.886(4)
Cu1 N2	1.979(2)	O15 H15B O8^1^	2.768(3)
Cu1 O3	2.156(2)	O15 H15A O2^2^	2.800(3)
Cu1 O1	2.170(2)	O14 H14B O6^3^	2.843(4)
		O14 H14B O8^1^	3.184(4)
Atom-Atom-Atom	Angle/°	O9 H9A O5	2.715(3)
N1 Cu1 N2	142.81(11)	O12 H12B O2^2^	2.840(3)
N1 Cu1 O3	97.54(9)	O12 H12A O7^1^	2.822(3)
N2 Cu1 O3	105.30(9)	O9 H9B O14^4^	2.774(4)
N1 Cu1 O1	120.81(9)	O10 H10B O15^4^	2.913(4)
N2 Cu1 O1	79.83(9)	C7 H7 O7	3.079(4)
O3 Cu1 O1	107.48(9)	C2 H2 O3	3.011(4)

^1^+X, 1/2-Y, 1/2 + Z;

^2^1-X, 1-Y, Z;

^3^2-X, 1/2 + Y, 1/2-Z;

^4^+X,−1 + Y, +Z.

**Figure 2:**
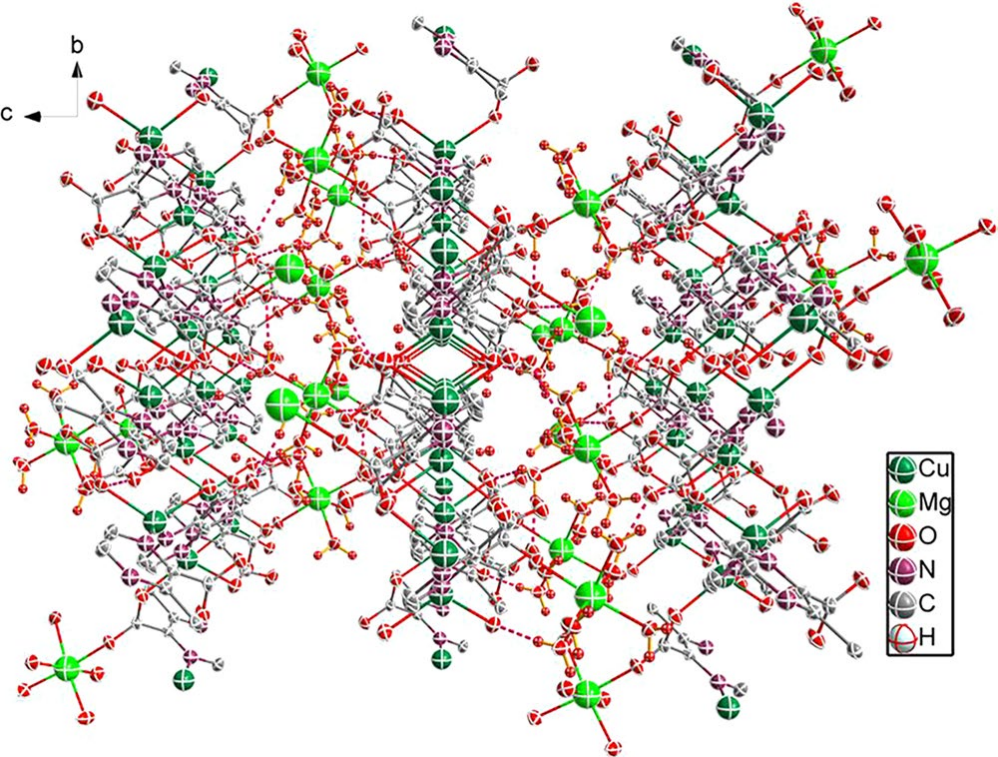
Schematic of hydrogen-bonding interaction.

There are intermolecular hydrogen-bonding interactions (e.g., O–H···O and C–H···O) between crystal water molecules and pzdc^2−^, coordination water molecules and pzdc^2−^ as well as crystal water molecules and coordination water molecules **(**Fig. 2**)**. The distances range from 2.656(3) to 3.298(4) Å. All the interactions promote the stability of the complex structure.

### Electrochemical measurement

**Figure 3:**
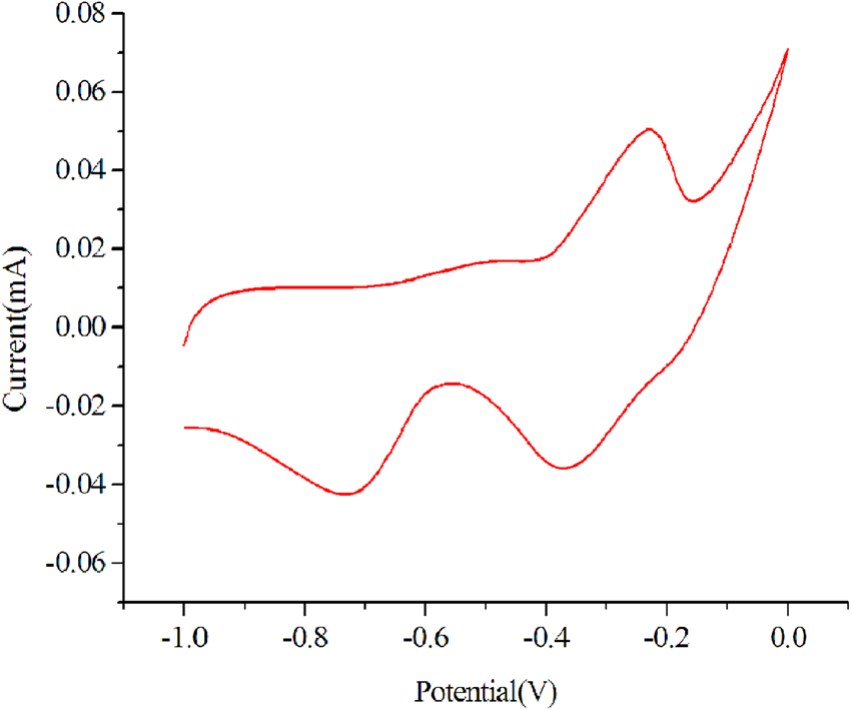
CV profile of Cu (I) complex at a potential scan rate of 50 mV s^−1^ in KOH solution of 1 mol L^−1^.

Cyclic voltammogram (CV) of the copper (I) complex was recorded at a potential scan rate of 50 mV s^−1^ in water solutions containing 1 mol L^−1^ of KOH **(**Fig. 3**)**. A conventional three-electrode arrangement was adopted, consisting of a glassy carbon as working electrode, saturated calomel as reference electrode, and a Pt wire as counter electrode. The sample was adhered to the working electrode. The CV reveals an incompletely reversible oxidation-reduction process in the “−1 to 0 V” range. Oxidation potential is observed at −0.233 V and reduction potential at −0.733 V and −0.373 V. It is inferred that the CV response is resulted from the following half reactions^33–35^:

1 $$[{{\rm{Cu}}}_{2}^{{\rm{I}}}{\rm{Mg}}{({\rm{pzdc}})}_{2}]\rightleftharpoons {[{{\rm{Cu}}}_{2}^{{\rm{I}}{\rm{I}}}{\rm{Mg}}{({\rm{pzdc}})}_{2}]}^{2+}+2{{\rm{e}}}^{-}$$

2 $${[{{\rm{Cu}}}_{2}^{{\rm{I}}{\rm{I}}}{\rm{Mg}}{({\rm{pzdc}})}_{2}]}^{2+}+{{\rm{e}}}^{-}\rightleftharpoons {[{{\rm{Cu}}}^{{\rm{I}}}{{\rm{Cu}}}^{{\rm{II}}}{\rm{Mg}}{({\rm{pzdc}})}_{2}]}^{+}$$

3 $${[{{\rm{Cu}}}^{{\rm{I}}}{{\rm{Cu}}}^{{\rm{II}}}{\rm{Mg}}{({\rm{pzdc}})}_{2}]}^{+}+{{\rm{e}}}^{-}\rightleftharpoons [{{\rm{Cu}}}_{2}^{{\rm{I}}}{\rm{Mg}}{({\rm{pzdc}})}_{2}]$$

On the oxidation scan, a single peak is observed, which is assigned to the complex two electrons oxidation half-reaction (Eq. 1). On the reduction scan, two well-defined peaks develop separated by about 360 mV, which show two stepwise reduction processes^36^ (Eqs. 2 and 3). The incompletely reversibility of the CV profile indicates that the mixed valence [Cu^I^Cu^II^(pzdc)_2_]^+^ and fully oxidized [Cu^II^Cu^II^Mg(pzdc)_2_]^2+^ intermediates maintain their integrity in the time scale of the CV experiment. We conclude that the oxidation of complex is Cu-centered which represents oxidation of Cu^+^ to its Cu^2+^ form.

### Photophysical properties

**Figure 4:**
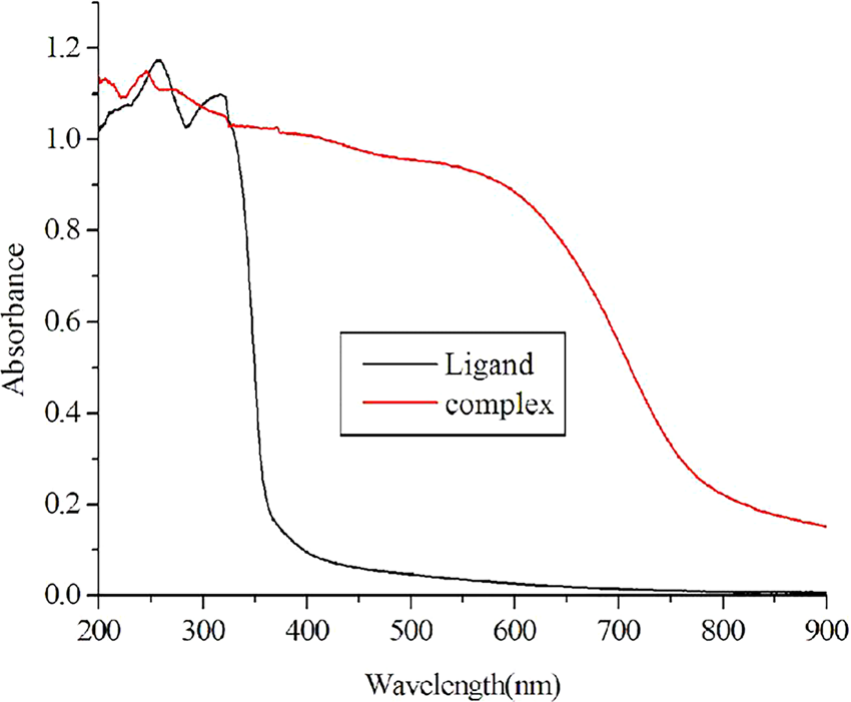
Absorption spectra of the complex and the free ligand in solid form.

The UV/Vis absorption spectra of free ligand 2,3-pyrazinedicarboxylic acid and copper (I) complex in solid form are shown in Fig. 4. The ligand shows intense absorption bands at 256–320 nm, which are assigned to the spin-allowed ligand-centered (LC) π–π* transition. As for the copper (I) complex, the intense absorption bands below 320 nm are also due to spin-allowed LC π–π* transition. In the 328–600 nm region, the copper (I) complex shows a broad shoulder, which can be attributed to metal-to-ligand charge-transfer (MLCT) transitions.

**Figure 5:**
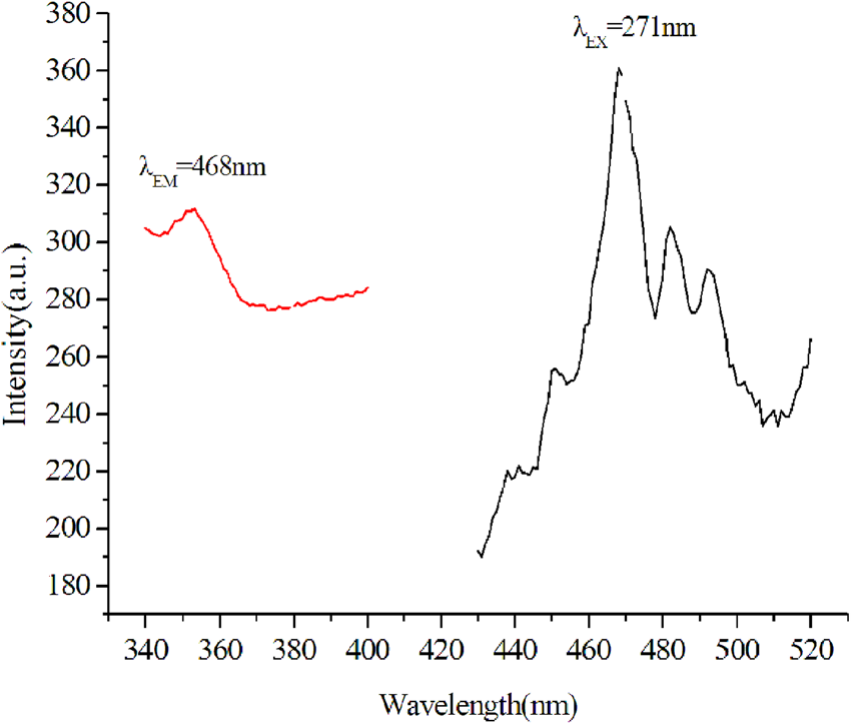
Photoluminescent excitation (EX) and emission (EM) spectra of the complex at room temperature.

The copper (I) complex exhibits blue photoluminescence emission (Fig. 5), which peaks at 468 nm. The emission is tentatively assigned to MLCT excited state rather than to cluster-centered (CC) triplet emission that consists of a combination of ligand-to-copper charge transfer (XMCT) and copper-centered d → s/p transitions^37^. It is mainly because the shortest Cu···Cu distance in the copper (I) complex is 4.369 Å, which is much larger than the sum of the vander Waals radii of Cu(I) (2.80 Å)^38–40^. This may be why the copper (I) complex does not display a remarkable photoluminescent property.

### Catalytic properties

**Figure 6:**
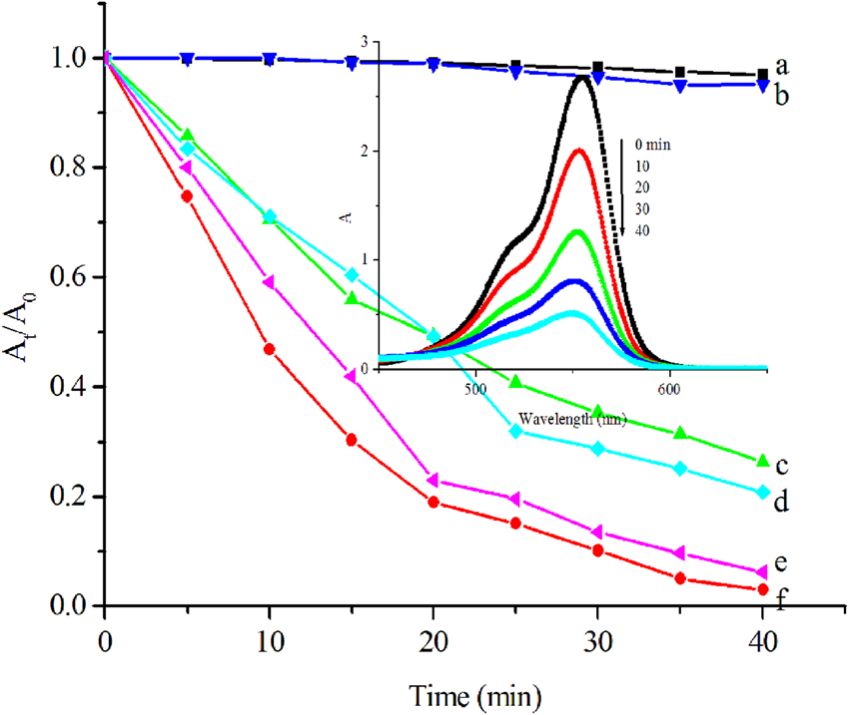
Degradation of RhB and MO (20 mg L^−1^): **(a,b**) without and (**e,f**) with Cu (I) complex in the presence of H_2_O_2_ and under visible light irradiation, (**c,d**) with Cu (I) complex in the presence of H_2_O_2_ but without visible light irradiation. Inset is the UV-Vis spectra of (**e**) versus irradiation time.

**Figure 7:**
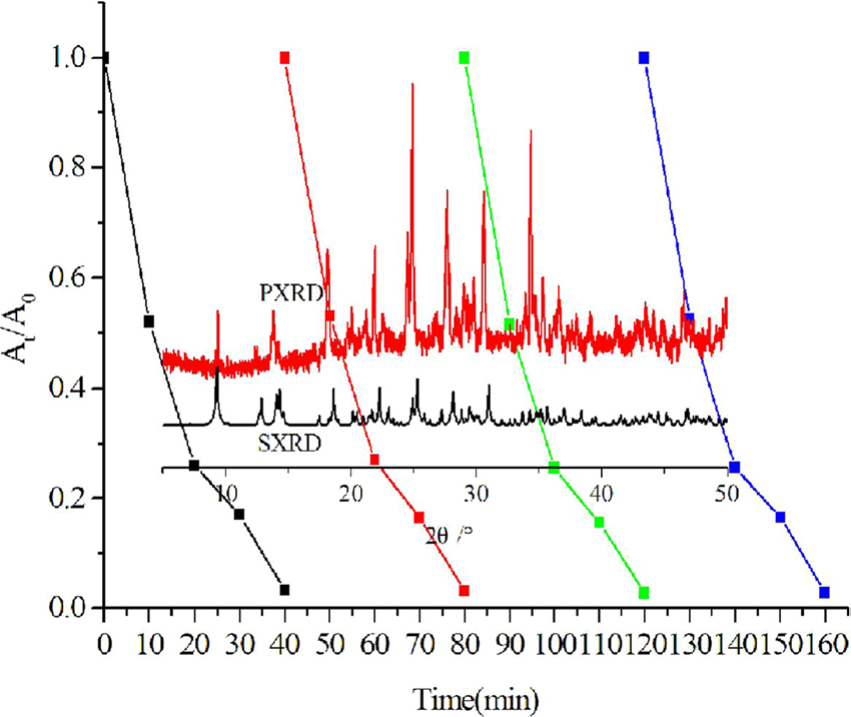
Cycling runs of glyphosate photodegradation under visible light irradiation and the PXRD and SXRD patterns of used catalyst after the four cycles (inset).

The photocatalytic performance of the copper (I) complex was tested for the degradation of RhB, MO and glyphosate under visible light irradiation **(**Fig. 6**)**. Without the copper (I) complex, there is insignificant degradation of RhB (curve **a**) and MO (curve **b**) in 40 min under visible light irradiation in the presence of H_2_O_2_. Under the same conditions but with the copper (I) complex, the degradation of RhB (curve **e** and inset) and MO (curve **f**) is over 96%. Without light irradiation, the degradation of the substrates substantially reduces (curve **c** for RhB and curve **d** for MO). To further evaluate the photocatalytic effect of copper (I) complex, the oxidation of glyphosate was carried out under similar conditions. Because glyphosate does not show any UV-Vis absorption, the degradation of glyphosate was monitored by TOC. There is no glyphosate conversion in the absence of copper (I) complex. In the presence of copper (I) complex, glyphosate was completely mineralized in 40 min **(**Fig. 7**)**. The results confirm that the copper (I) complex plays an important role in promoting the degradation of RhB, MO and glyphosate. Comparatively speaking, the degradation rate caused by copper (I) complex is faster than that caused by Cu_2_O nanoparticles, P25 or Cu_2_O-RGO-3 nanocomposite^41^, which degrade pollutants over 2 hours under the same conditions. We conclude that the catalytic systems must possess three factors including: oxidant, catalyst, and light. When visible light is introduced to a turbid liquid system containing RhB (MO or glyphosate)/Cu (I)/H_2_O_2_, the degradation rate of pollutants is faster than the reaction undergo without light irradiation, because visible light supplies much higher energy for the reaction. All the results suggest that H_2_O_2_, as oxidant, complex Cu (I), as catalyst, play an important role in the degradation rate of the pollutants, and light energy accelerates the degradation of pollutants.

Considering reusability is an essential factor for the application of a photocatalyst, we tested the recyclability of copper (I) complex in four cycles of glyphosate degradation **(**Fig. 7**)**. The results show that there is no significant decrease of degradation efficiency across the four runs. Moreover, the PXRD pattern of the used sample is similar to that of the fresh sample (Fig. 7, inset), revealing that the catalyst is photochemically stable during the degradation process.

### Catalytic mechanism

**Figure 8:**
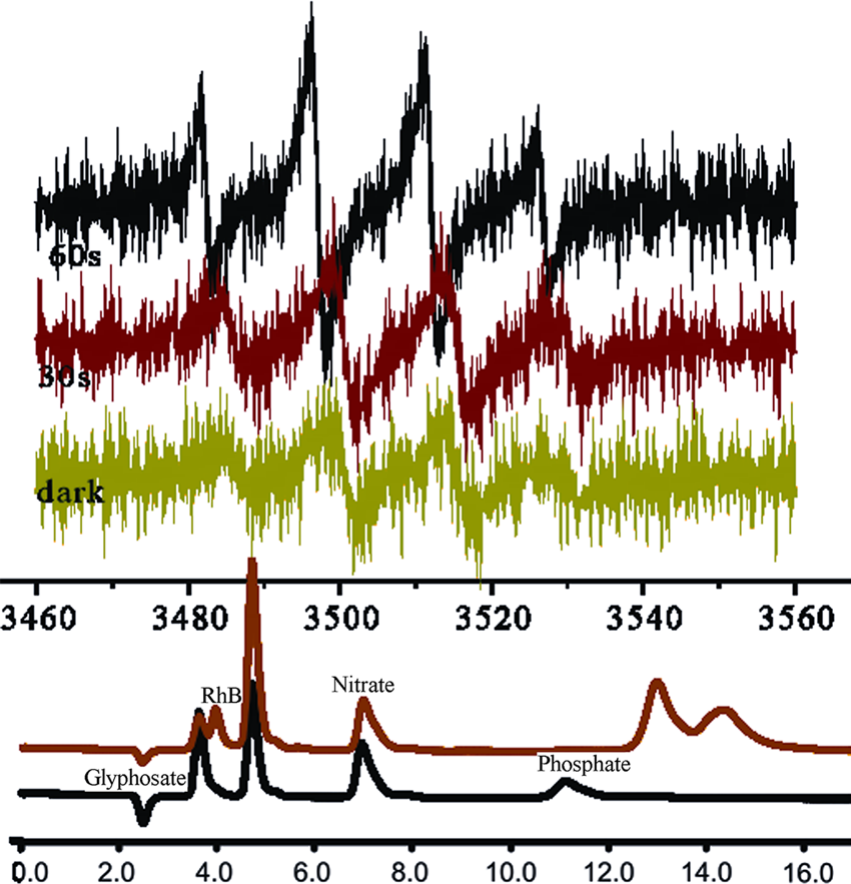
(Top) DMPO spin-trapping ESR spectrum of RhB aqueous solution in the presence of Cu (I) complex under visible light irradiation as well as that in the dark, and (Bottom) IC results of RhB and glyphosate degradation.

ESR technique was used to capture short-lived radicals during the degradation process under darkness and visible light irradiation conditions. As shown in Fig. 8, signals of 5,5-dimethyl-1-pyrroline-N-oxide (DMPO)/ ∙ OH adducts are clearly observed in the dark as well as under visible light irradiation, displaying the characteristic intensity ratio of 1:2:2:1(hyperfine coupling constants α_H_ = α _N_ = 14.9 G)^42,43^. With increase of light absorption, there is increase of signal intensity (from 30 to 60 s). Nonetheless, the signal intensity is much smaller in the dark, implying that the degradation rate is slower in the absence of visible light irradiation. In addition, the corresponding inorganic ions (NO_3_^−^ and PO_4_^3−^) were detected by ion chromatography. These findings provide direct evidence for the participation of ∙OH radicals in the photocatalytic processes. It is inferred that the ∙OH radicals of high oxidation potential facilitate the degradation, and with visible light absorption, the degradation of the dyes and glyphosate is accelerated.

The degradation mechanism is complicated. It is difficult to monitor the decomposed intermediate compounds because of the rapid degradation. Based on the change of ∙OH radical and TOC content, as well as the information related to N and P products and CV curves, it is inferred that ∙OH radicals are dominant active species in the photocatalytic processes^44–46^. At first, the [Cu_2_^I^Mg(pzdc)_2_] catalyst causes the decomposition of H_2_O_2_ and the formation of ∙OH radicals, with itself converted to [Cu^I^Cu^II^Mg(pzdc)_2_]^+^ or [Cu_2_^II^Mg(pzdc)_2_]^2+^. Subsequently, the reduction of [Cu^I^Cu^II^Mg(pzdc)_2_]^+^ or [Cu_2_^II^Mg(pzdc)_2_]^2+^ results in the production of ∙OH radicals as well as the regeneration of [Cu_2_^I^Mg(pzdc)_2_]. Consequently, there is a catalytic cycle of Cu oxidation states between [Cu_2_^I^Mg(pzdc)_2_] and [Cu^I^Cu^II^Mg(pzdc)_2_]^+^ or [Cu_2_^II^Mg(pzdc)_2_]^2+^. Overall, under visible light irradiation and with the absorption of light energy, there is the fast degradation of dyes and glyphosate by ∙OH radicals under the cooperative influence of [Cu_2_^I^Mg(pzdc)_2_] and H_2_O_2_.

## Conclusion

In summary, a new cuprous polymer with 2,3-pyrazinedicarboxylic acid ligands was synthesized by hydrothermal method. It is noteworthy that the central Cu^+^ ions are in *situ* redox reaction. The polymer possesses photoluminescence property, exhibiting emission behavior similar to that observed in solid state. In addition, the polymer shows effective catalytic activity in the degradation of dyes and glyphosate under moderate reaction conditions using H_2_O_2_ as green oxidant. The polymer can be easily recovered and reused in these heterogeneous catalytic systems without significant loss of catalytic activity. The plausible catalytic mechanism suggests the generation of ∙OH radicals and active copper intermediates that are responsible for the degradation of the substrates. Based on the outstanding performance of the polymer, it is envisaged that it has high application potentials in the field of catalysis.
